# (*E*)-4-[(3,5-Dimethyl­phen­yl)imino­meth­yl]-2-meth­oxy-3-nitro­phenol

**DOI:** 10.1107/S1600536811054407

**Published:** 2011-12-23

**Authors:** Zhong-Ming Yang, Deng-Cheng Su, Hai-Liang Zhu

**Affiliations:** a School of Life Sciences, Shangdong University of Technology, Zibo 255000, People’s Republic of China

## Abstract

The mol­ecule of the title compound, C_16_H_16_N_2_O_4_, exists in the *E* configuration with respect to the central C=N double bond. The dihedral angle between the two benzene rings is 2.17 (9) Å. In the crystal, mol­ecules are linked *via* O—H⋯N hydrogen bonds into chains that propagate along the *b*-axis direction. There is also π–π stacking of inversion-related mol­ecules, with inter­planar spacings of 3.479 (5) Å and ring centroid–centroid distances of 3.876 (4) Å.

## Related literature

The title compound is an imine derivative of 4-hy­droxy-3-meth­oxy-2-nitro­benzaldehyde, a vanillin-like compound. For background to the biological activity of vanillin derivatives, see: Javiya *et al.* (2008 ▶); Cordano *et al.* (2002 ▶). For standard bond lengths, see: Allen *et al.* (1987 ▶).
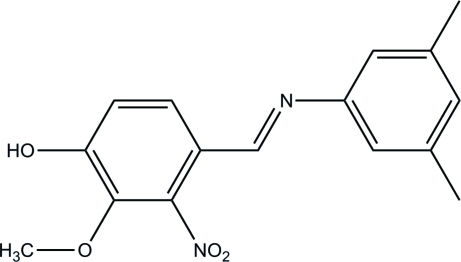


## Experimental

### 

#### Crystal data

C_16_H_16_N_2_O_4_
*M* *_r_* = 300.31
Monoclinic, 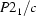

*a* = 8.616 (9) Å
*b* = 9.690 (11) Å
*c* = 18.29 (2) Å
β = 97.631 (11)°
*V* = 1513 (3) Å^3^
*Z* = 4
Mo *K*α radiation
μ = 0.10 mm^−1^
*T* = 296 K
0.23 × 0.21 × 0.12 mm

#### Data collection

Bruker APEXII CCD diffractometer
Absorption correction: multi-scan (*SADABS*; Bruker, 2007 ▶) *T*_min_ = 0.978, *T*_max_ = 0.989
8085 measured reflections
2935 independent reflections
1699 reflections with *I* > 2σ(*I*)
*R*_int_ = 0.032

#### Refinement

*R*[*F* ^2^ > 2σ(*F* ^2^)] = 0.046
*wR*(*F* ^2^) = 0.140
*S* = 1.04
2935 reflections
203 parameters
H-atom parameters constrained
Δρ_max_ = 0.24 e Å^−3^
Δρ_min_ = −0.17 e Å^−3^

### 

Data collection: *APEX2* (Bruker, 2007 ▶); cell refinement: *SAINT* (Bruker, 2007 ▶); data reduction: *SAINT*; program(s) used to solve structure: *SHELXS97* (Sheldrick, 2008 ▶); program(s) used to refine structure: *SHELXL97* (Sheldrick, 2008 ▶); molecular graphics: *SHELXTL* (Sheldrick, 2008 ▶); software used to prepare material for publication: *SHELXTL*.

## Supplementary Material

Crystal structure: contains datablock(s) global, I. DOI: 10.1107/S1600536811054407/pk2377sup1.cif

Structure factors: contains datablock(s) I. DOI: 10.1107/S1600536811054407/pk2377Isup2.hkl

Supplementary material file. DOI: 10.1107/S1600536811054407/pk2377Isup3.cml

Additional supplementary materials:  crystallographic information; 3D view; checkCIF report

## Figures and Tables

**Table 1:** Hydrogen-bond geometry (Å, °)

*D*—H⋯*A*	*D*—H	H⋯*A*	*D*⋯*A*	*D*—H⋯*A*
O2—H2⋯N2^i^	0.82	1.95	2.755 (3)	169

Symmetry code: (i) 
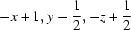
.

**Fig. 1.**
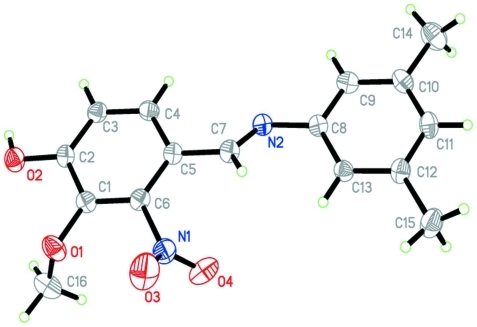
The molecular structure of (I) showing 30% probability displacement ellipsoids.

## supplementary crystallographic information

### Comment

The Schiff base adducts of vanillin with various primary amines show an observable effect on tumours.  Amine derivatives of vanillin present similar effects, such as acting as oxidative phosphorylation inhibitors, which prevent ATP sythesis (Cordano *et al.*, 2002).  There has been much research interest in vanillin derivatives due to its biological activities (Javiya *et al.* 2008). In this work, we report here the crystal structure of the title compound, (*E*)-4-((3,5-dimethylphenylimino)methyl)-2-methoxy-3-nitrophenol, (I). In (I), all bond lengths are within normal ranges (Allen *et al.*, 1987) (Fig. 1).

### Experimental

An equimolar ratio of 4-hydroxy-3-methoxy-2-nitrobenzaldehyde (2 mmol, 394 mg) and 3,5-dimethylaniline (2 mmol, 242 mg) were dissolved in methanol (25 ml). The mixture was stirred for 3 h under a gentle reflux.  After allowing the solution to stand in air for two days with methanol slowly evaporating, yellow crystals were deposited, isolated, washed with methanol three times, and dried under vacuum with CaCl_2_ as desiccant.

### Refinement

All H atoms were positioned geometrically (C—H = 0.96 Å for the aromatic) and were refined as riding, with *U*_iso_(H) = 1.2*U*_eq_(C) and *U*_iso_(H) = 1.2*U*_eq_(N).

### Crystal data

**Table:**

C_16_H_16_N_2_O_4_	*F*(000) = 632
*M**_r_* = 300.31	*D*_x_ = 1.318 Mg m^−^^3^
Monoclinic, *P*2_1_/*c*	Mo *K*α radiation, λ = 0.71073 Å
Hall symbol: -P 2ybc	Cell parameters from 2115 reflections
*a* = 8.616 (9) Å	θ = 2.4–26.4°
*b* = 9.690 (11) Å	µ = 0.10 mm^−^^1^
*c* = 18.29 (2) Å	*T* = 296 K
β = 97.631 (11)°	Column, yellow
*V* = 1513 (3) Å^3^	0.23 × 0.21 × 0.12 mm
*Z* = 4	

### Data collection

**Table:**

Bruker APEXII CCD diffractometer	2935 independent reflections
Radiation source: fine-focus sealed tube	1699 reflections with *I* > 2σ(*I*)
graphite	*R*_int_ = 0.032
φ and ω scans	θ_max_ = 26.0°, θ_min_ = 2.4°
Absorption correction: multi-scan (*SADABS*; Bruker, 2007)	*h* = −8→10
*T*_min_ = 0.978, *T*_max_ = 0.989	*k* = −11→11
8085 measured reflections	*l* = −16→22

### Refinement

**Table:**

Refinement on *F*^2^	Primary atom site location: structure-invariant direct methods
Least-squares matrix: full	Secondary atom site location: difference Fourier map
*R*[*F*^2^ > 2σ(*F*^2^)] = 0.046	Hydrogen site location: inferred from neighbouring sites
*wR*(*F*^2^) = 0.140	H-atom parameters constrained
*S* = 1.04	*w* = 1/[σ^2^(*F*_o_^2^) + (0.0684*P*)^2^ + 0.080*P*] where *P* = (*F*_o_^2^ + 2*F*_c_^2^)/3
2935 reflections	(Δ/σ)_max_ < 0.001
203 parameters	Δρ_max_ = 0.24 e Å^−^^3^
0 restraints	Δρ_min_ = −0.17 e Å^−^^3^

### Special details

**Table:**

Geometry. All e.s.d.'s (except the e.s.d. in the dihedral angle between two l.s. planes) are estimated using the full covariance matrix. The cell e.s.d.'s are taken into account individually in the estimation of e.s.d.'s in distances, angles and torsion angles; correlations between e.s.d.'s in cell parameters are only used when they are defined by crystal symmetry. An approximate (isotropic) treatment of cell e.s.d.'s is used for estimating e.s.d.'s involving l.s. planes.
Refinement. Refinement of *F*^2^ against ALL reflections. The weighted *R*-factor *wR* and goodness of fit *S* are based on *F*^2^, conventional *R*-factors *R* are based on *F*, with *F* set to zero for negative *F*^2^. The threshold expression of *F*^2^ > σ(*F*^2^) is used only for calculating *R*-factors(gt) *etc*. and is not relevant to the choice of reflections for refinement. *R*-factors based on *F*^2^ are statistically about twice as large as those based on *F*, and *R*- factors based on ALL data will be even larger.

### Fractional atomic coordinates and isotropic or equivalent isotropic displacement parameters (Å^2^)

**Table:**

	*x*	*y*	*z*	*U*_iso_*/*U*_eq_	
N1	0.9369 (2)	0.4869 (2)	0.39259 (10)	0.0569 (5)	
N2	0.50451 (19)	0.63346 (16)	0.41616 (8)	0.0416 (4)	
O1	0.98163 (17)	0.29564 (16)	0.28424 (8)	0.0592 (4)	
O2	0.73798 (16)	0.18585 (15)	0.19443 (8)	0.0542 (4)	
H2	0.6585	0.1695	0.1658	0.081*	
O3	1.0325 (2)	0.4118 (2)	0.42663 (10)	0.0972 (7)	
O4	0.9417 (2)	0.61258 (19)	0.39560 (10)	0.0811 (6)	
C1	0.8322 (2)	0.33311 (19)	0.29183 (11)	0.0410 (5)	
C2	0.7044 (2)	0.27628 (19)	0.24588 (10)	0.0401 (5)	
C3	0.5541 (2)	0.3148 (2)	0.25587 (11)	0.0441 (5)	
H3	0.4693	0.2778	0.2254	0.053*	
C4	0.5275 (2)	0.4069 (2)	0.31015 (11)	0.0428 (5)	
H4	0.4251	0.4309	0.3155	0.051*	
C5	0.6504 (2)	0.46491 (19)	0.35722 (10)	0.0395 (5)	
C6	0.8019 (2)	0.42449 (19)	0.34604 (10)	0.0404 (5)	
C7	0.6243 (2)	0.5559 (2)	0.41790 (11)	0.0427 (5)	
H7	0.6985	0.5573	0.4598	0.051*	
C8	0.4898 (2)	0.72319 (19)	0.47734 (10)	0.0389 (5)	
C9	0.3404 (2)	0.7652 (2)	0.48727 (11)	0.0452 (5)	
H9	0.2545	0.7324	0.4559	0.054*	
C10	0.3177 (2)	0.8561 (2)	0.54361 (12)	0.0473 (6)	
C11	0.4476 (3)	0.9040 (2)	0.58903 (11)	0.0492 (6)	
H11	0.4334	0.9655	0.6266	0.059*	
C12	0.5983 (2)	0.86339 (19)	0.58038 (11)	0.0452 (5)	
C13	0.6190 (2)	0.77377 (19)	0.52390 (11)	0.0409 (5)	
H13	0.7195	0.7469	0.5168	0.049*	
C14	0.1558 (3)	0.9054 (3)	0.55369 (15)	0.0742 (8)	
H14A	0.0792	0.8536	0.5220	0.111*	
H14B	0.1460	1.0016	0.5414	0.111*	
H14C	0.1392	0.8924	0.6041	0.111*	
C15	0.7379 (3)	0.9183 (2)	0.63073 (13)	0.0659 (7)	
H15A	0.8273	0.8609	0.6269	0.099*	
H15B	0.7153	0.9180	0.6807	0.099*	
H15C	0.7599	1.0109	0.6165	0.099*	
C16	1.0488 (3)	0.3650 (3)	0.22692 (16)	0.0830 (8)	
H16A	0.9857	0.3485	0.1804	0.124*	
H16B	1.1528	0.3308	0.2251	0.124*	
H16C	1.0531	0.4624	0.2368	0.124*	

### Atomic displacement parameters (Å^2^)

**Table:**

	*U*^11^	*U*^22^	*U*^33^	*U*^12^	*U*^13^	*U*^23^
N1	0.0502 (12)	0.0687 (13)	0.0484 (12)	0.0024 (10)	−0.0067 (9)	−0.0119 (10)
N2	0.0453 (10)	0.0436 (9)	0.0355 (10)	0.0019 (8)	0.0038 (8)	0.0029 (8)
O1	0.0433 (9)	0.0764 (11)	0.0564 (10)	0.0165 (8)	0.0017 (7)	−0.0015 (8)
O2	0.0520 (9)	0.0614 (9)	0.0468 (10)	0.0059 (8)	−0.0026 (7)	−0.0146 (8)
O3	0.0803 (14)	0.1037 (15)	0.0914 (14)	0.0299 (11)	−0.0490 (11)	−0.0251 (12)
O4	0.0812 (13)	0.0707 (12)	0.0860 (14)	−0.0246 (10)	−0.0088 (10)	−0.0067 (10)
C1	0.0394 (12)	0.0467 (11)	0.0355 (12)	0.0061 (9)	−0.0004 (9)	0.0031 (9)
C2	0.0482 (13)	0.0393 (10)	0.0317 (11)	0.0046 (9)	0.0012 (9)	−0.0001 (9)
C3	0.0433 (12)	0.0484 (12)	0.0390 (12)	−0.0026 (9)	−0.0005 (9)	−0.0031 (10)
C4	0.0380 (12)	0.0462 (11)	0.0436 (13)	0.0011 (9)	0.0029 (10)	0.0034 (10)
C5	0.0449 (13)	0.0393 (10)	0.0332 (11)	0.0033 (9)	0.0016 (9)	0.0023 (9)
C6	0.0415 (12)	0.0442 (11)	0.0324 (11)	0.0007 (9)	−0.0067 (9)	0.0010 (9)
C7	0.0474 (13)	0.0447 (11)	0.0342 (12)	0.0026 (10)	−0.0010 (9)	0.0020 (9)
C8	0.0443 (12)	0.0382 (10)	0.0339 (12)	0.0043 (9)	0.0037 (9)	0.0060 (9)
C9	0.0410 (13)	0.0492 (12)	0.0436 (13)	0.0058 (9)	−0.0014 (10)	0.0100 (10)
C10	0.0481 (13)	0.0490 (12)	0.0460 (13)	0.0147 (10)	0.0102 (11)	0.0100 (11)
C11	0.0618 (16)	0.0423 (11)	0.0438 (13)	0.0138 (10)	0.0080 (11)	−0.0011 (10)
C12	0.0502 (13)	0.0399 (11)	0.0434 (13)	0.0099 (9)	−0.0009 (10)	−0.0005 (10)
C13	0.0362 (12)	0.0418 (10)	0.0443 (12)	0.0075 (8)	0.0039 (9)	0.0000 (9)
C14	0.0547 (16)	0.0839 (18)	0.085 (2)	0.0269 (13)	0.0137 (14)	0.0051 (15)
C15	0.0650 (16)	0.0637 (15)	0.0644 (16)	0.0076 (12)	−0.0091 (13)	−0.0172 (12)
C16	0.0587 (17)	0.101 (2)	0.093 (2)	0.0072 (15)	0.0242 (16)	0.0017 (18)

### Geometric parameters (Å, °)

**Table:**

N1—O3	1.208 (2)	C8—C13	1.398 (3)
N1—O4	1.219 (3)	C9—C10	1.389 (3)
N1—C6	1.476 (3)	C9—H9	0.9300
N2—C7	1.274 (3)	C10—C11	1.383 (3)
N2—C8	1.436 (3)	C10—C14	1.509 (3)
O1—C1	1.362 (3)	C11—C12	1.385 (3)
O1—C16	1.431 (3)	C11—H11	0.9300
O2—C2	1.345 (2)	C12—C13	1.379 (3)
O2—H2	0.8200	C12—C15	1.512 (3)
C1—C6	1.380 (3)	C13—H13	0.9300
C1—C2	1.406 (3)	C14—H14A	0.9600
C2—C3	1.383 (3)	C14—H14B	0.9600
C3—C4	1.377 (3)	C14—H14C	0.9600
C3—H3	0.9300	C15—H15A	0.9600
C4—C5	1.392 (3)	C15—H15B	0.9600
C4—H4	0.9300	C15—H15C	0.9600
C5—C6	1.404 (3)	C16—H16A	0.9600
C5—C7	1.458 (3)	C16—H16B	0.9600
C7—H7	0.9300	C16—H16C	0.9600
C8—C9	1.385 (3)		
			
O3—N1—O4	124.1 (2)	C10—C9—H9	119.7
O3—N1—C6	118.8 (2)	C11—C10—C9	118.4 (2)
O4—N1—C6	117.06 (19)	C11—C10—C14	120.5 (2)
C7—N2—C8	119.54 (17)	C9—C10—C14	121.0 (2)
C1—O1—C16	115.41 (18)	C10—C11—C12	122.2 (2)
C2—O2—H2	109.5	C10—C11—H11	118.9
O1—C1—C6	121.05 (17)	C12—C11—H11	118.9
O1—C1—C2	120.70 (19)	C13—C12—C11	118.75 (19)
C6—C1—C2	118.22 (19)	C13—C12—C15	120.4 (2)
O2—C2—C3	124.08 (18)	C11—C12—C15	120.8 (2)
O2—C2—C1	116.69 (19)	C12—C13—C8	120.32 (19)
C3—C2—C1	119.23 (19)	C12—C13—H13	119.8
C4—C3—C2	121.24 (18)	C8—C13—H13	119.8
C4—C3—H3	119.4	C10—C14—H14A	109.5
C2—C3—H3	119.4	C10—C14—H14B	109.5
C3—C4—C5	121.47 (19)	H14A—C14—H14B	109.5
C3—C4—H4	119.3	C10—C14—H14C	109.5
C5—C4—H4	119.3	H14A—C14—H14C	109.5
C4—C5—C6	116.32 (19)	H14B—C14—H14C	109.5
C4—C5—C7	122.25 (19)	C12—C15—H15A	109.5
C6—C5—C7	121.32 (18)	C12—C15—H15B	109.5
C1—C6—C5	123.51 (18)	H15A—C15—H15B	109.5
C1—C6—N1	117.89 (19)	C12—C15—H15C	109.5
C5—C6—N1	118.56 (19)	H15A—C15—H15C	109.5
N2—C7—C5	122.96 (18)	H15B—C15—H15C	109.5
N2—C7—H7	118.5	O1—C16—H16A	109.5
C5—C7—H7	118.5	O1—C16—H16B	109.5
C9—C8—C13	119.75 (19)	H16A—C16—H16B	109.5
C9—C8—N2	117.40 (18)	O1—C16—H16C	109.5
C13—C8—N2	122.77 (18)	H16A—C16—H16C	109.5
C8—C9—C10	120.6 (2)	H16B—C16—H16C	109.5
C8—C9—H9	119.7		

### Hydrogen-bond geometry (Å, °)

**Table:**

*D*—H···*A*	*D*—H	H···*A*	*D*···*A*	*D*—H···*A*
O2—H2···N2^i^	0.82	1.95	2.755 (3)	169.

Symmetry codes: (i) −*x*+1, *y*−1/2, −*z*+1/2.
